# The evolution and anatomy of the horse manus with an emphasis on digit reduction

**DOI:** 10.1098/rsos.171782

**Published:** 2018-01-24

**Authors:** Nikos Solounias, Melinda Danowitz, Elizabeth Stachtiaris, Abhilasha Khurana, Marwan Araim, Marc Sayegh, Jessica Natale

**Affiliations:** 1 Department of Anatomy, New York Institute of Technology College of Osteopathic Medicine, 8000 Northern Boulevard, Old Westbury, NY 11568, USA; 2 Department of Paleontology, American Museum of Natural History, Central Park West at 79th Street, New York, NY 10024, USA; 3 Department of Pediatrics, Alfred I. duPont Hospital for Children, 1600 Rockland Road, Wilmington, DE 19803, USA; 4 Department of Emergency Medicine, Brookdale University Hospital and Medical Center, 1 Brookdale Plaza, Brooklyn, NY 11212, USA; 5 Department of Internal Medicine, Saint Michael's Medical Center, 111 Central Avenue, Newark, NJ 07102, USA

**Keywords:** horse, *Equus*, forelimb, monodactyl, digit reduction

## Abstract

We revisit digit reduction in the horse and propose that all five digits are partially present in the modern adult forelimb. Osteological descriptions of selected tetradactyl, tridactyl and monodactyl equids demonstrate the evolution of the forelimb. Histological, osteological and palaeontological evidence suggest that the *Equus* distal forelimb is more complex than traditionally conceived. The current understanding is that the horse distal forelimb consists of one complete digit (III) and two reduced splint metacarpals (II and IV). Metacarpals II and IV each exhibit a ventral ridge, which we suggest represents the undifferentiated digits I and V. These ridges are present in the tridactyl *Mesohippus*, but are absent in the tetradactyl *Hyracotherium.* The carpal articulations of the five metacarpals match those of pentadactyl taxa. Distally, the frog, a V-shaped structure on the ventral hoof represents digits II and IV, and the wings and hoof cartilages of the distal phalanx are digits I and V. We relate this revised interpretation of the *Equus* forelimb to Laetoli footprints, and suggest the *Hipparion* side impressions are created from the hooves of I and V, rather than from II and IV. We show shades of pentadactyly within the *Equus* manus.

## 1. Introduction

Digit reduction is common among mammals. In contrast to the squamates, where entire limbs are lost completely, mammals exhibit a lesser degree of reduction where only individual digits are lost or simplified. In many carnivores, digit I is removed from the locomotory apparatus, but persists on the limb as the dewclaw [1]. Among the artiodactyls, digits III and IV are fused centrally, digits II and V are reduced into smaller side toes, and I is often entirely eliminated [2]. The reduction of the side toes in artiodactyls is variable. For example, in cervids, splint metacarpals and external hooves are present, but the side toes lack phalanges, whereas in suids, the side toes are complete but small [3]. Within the mani of perissodactyls other than the horse, rhinoceroses possess three fully formed digits and tapirs have four complete digits [4].

The horse lineage exhibits the most extreme digit reduction, resulting in the monodactyl forelimb of *Equus*. *Hyracotherium* at the onset of the equid evolution had four metacarpals. The common subsequent main taxon is *Mesohippus* where the digits are reduced to three. *Hyracotherium* gave rise to the numerous tridactyl equids that prevailed from the Early Oligocene to the Pleistocene [5–7]. The adaptive radiations of equids were pervasive throughout North America and the Old World. Ultimately, certain tridactyl equids gave rise to the modern horse, whose limb is composed of a single complete digit (III) with reduced splint digits (II and IV).

Monodactyly occurred twice within the equid lineages. The first time it occurred was in the Miocene, starting with *Dinohippus*, leading to the modern horse [6]. Monodactyly also occurred by convergent evolution in the lineage to *Pliohippus* and *Astrohippus*, single-toed horses that are now extinct [6]. The reduction to a single complete toe also evolved in the liptotern *Thoatherium*, an extinct ungulate from the Miocene of South America [5].

The reduction of digits in the horse is accompanied by an increase in overall limb length [8], therefore increasing the distance of each stride. Janis [6] proposed that monodactyly evolves to allow the trot gait characteristic of the modern horse. The limb adapted for the faster trot gait facilitates locomotion in the grassland habitat, as horses are known grazers [6]. The horse limb evolved to move primarily in flexion and extension, and the overall limb structure prevents supination and pronation [8]. In addition, the simplification of the horse hand into a single complete digit stabilizes the limb by reducing the total number of joints.

In this study, we use the term *Equus* for the domesticated horse *Equus caballus*. We suggest changing the identity of certain structures of the manus (distal forelimb) of the domesticated horse. Using the osteology, joint articulations, nerve and vessel distribution, and palaeontology of the distal forelimb, we theorize that the *Equus* manus maintains remnants of the ‘missing’ digits. While it is already known that digits II and IV persist proximally as the splint metacarpals, we propose that digits I and V are also present proximally and that components of all five digits are found distally within the manus.

We show that the evolutionary change to monodactyly is not as dramatic as previously thought and that the horse forelimb is more similar to that of its pentadactyl, tetradactyl and tridactyl ancestors. Although the modern horse maintains only one complete digit, the identities of all five digits are preserved in both the skeletal and soft anatomy as embedded elements into the dominant digit, and the digit positions are consistent with horses in earlier stages of evolution. We propose an hourglass pattern of reduction where the proximal- and distal-most parts of the digits are retained, the middle regions of the digits are completely reduced. We question the degree of digit reduction in both extinct and extant horses, and rethink the status quo of monodactyly in *Equus*. Our study suggests elements of pentadactyly among the most famous single-toed species.

## 2. Material and methods

Two *Equus* fetal specimens were obtained from Ward's Natural Science pre-fixed in formalin and frozen. The gestational ages of the fetal specimens were estimated using the femoral length [9] and approximate crown–rump length [10]. The extremities of these fetal specimens were removed and sliced in transverse sections from the proximal metacarpal to the distal phalanx using a band saw, creating five sections. These sections include proximal end of metacarpal III, distal end of metacarpal III near the termination of metacarpals II and IV, proximal end of the proximal phalanx, proximal end of the middle phalanx, and proximal end of the distal phalanx. Thin slices of these sections were stained using haematoxylin and eosin (H&E) stain using standard protocols, creating microscope slides. These slides were examined at 40× magnification on a standard light microscope for normal histological structures. Using these slides, we counted the number of large nerve bundles and arteries from the five sections, to map the neurovascular distribution on the limb. We recorded all nerve bundles and arteries that were greater than 0.4 mm in size. We mapped the nerves and arteries on two slides per gross section to confirm the number and position of nerves and arteries in each region.

The hoof of a full-term *Equus* specimen was cut in three coronal sections perpendicular to the axis of the distal phalanx, creating three sections. These sections include the proximal end of the distal phalanx including the hoof cartilages, middle end of the distal phalanx including the frog, and distal end of the distal phalanx. An additional adult *Equus* hoof was sectioned in two coronal cuts, creating two sections. These sections include the proximal end of the distal phalanx and middle end of the distal phalanx near the merging of the frog. These three full-term sections and two adult sections were used to examine the gross morphology of the frog and keratinous hoof.

We describe the detailed osteology and metacarpal articulations of the horse and several extinct equids using specimens from the American Museum of Natural History, Yale Peabody Museum and Museum of Comparative Zoology at Harvard. From the line of taxa leading to the modern horse, we selected a representative tetradactyl (*Hyracotherium*), tridactyl (*Mesohippus*) and monodactyl (*Dinohippus*) equid, as well as the modern horse to delineate the osteology and carpal–metacarpal articulations.

We chose *Hyracotherium* as the tetradactyl horse as this represents the onset of the lineage of equids. *Mesohippus* was chosen as this is an abundant taxon and represents one of the first tridactyl horses. *Dinohippus* represents the direct ancestor of *Equus*.

We also describe the articulations of a representative pentadactyl (*Phenacodus*). This taxon was chosen as the pentadactyl species as this is a condylarth that is close to the origin of the ungulates. We also describe the proximal carpal–metacarpal morphology of a tridactyl *Hypohippus* that has a unique specialization among equids. We also describe the *Hipparion* footprints from Laetoli and the osteology of the distal limb of this taxon.

## 3. Results

### 3.1. Ages of the fetal horse specimens

Using the crown–rump length [10] and ossified femoral length [9], we approximate the gestational age of our fetal specimens as the following:

NS 290: unossified femoral length: 64 mm (gestational age: 185 days); crown–rump: 58 cm (gestational age: 180–199 days);
NS 291: no data;
NS 292: unossified femoral length: 69 mm (gestational age: 193 days); crown–rump: 58 cm (gestational age: 180–199 days).

### 3.2. Phylogeny

**Figure 1.**
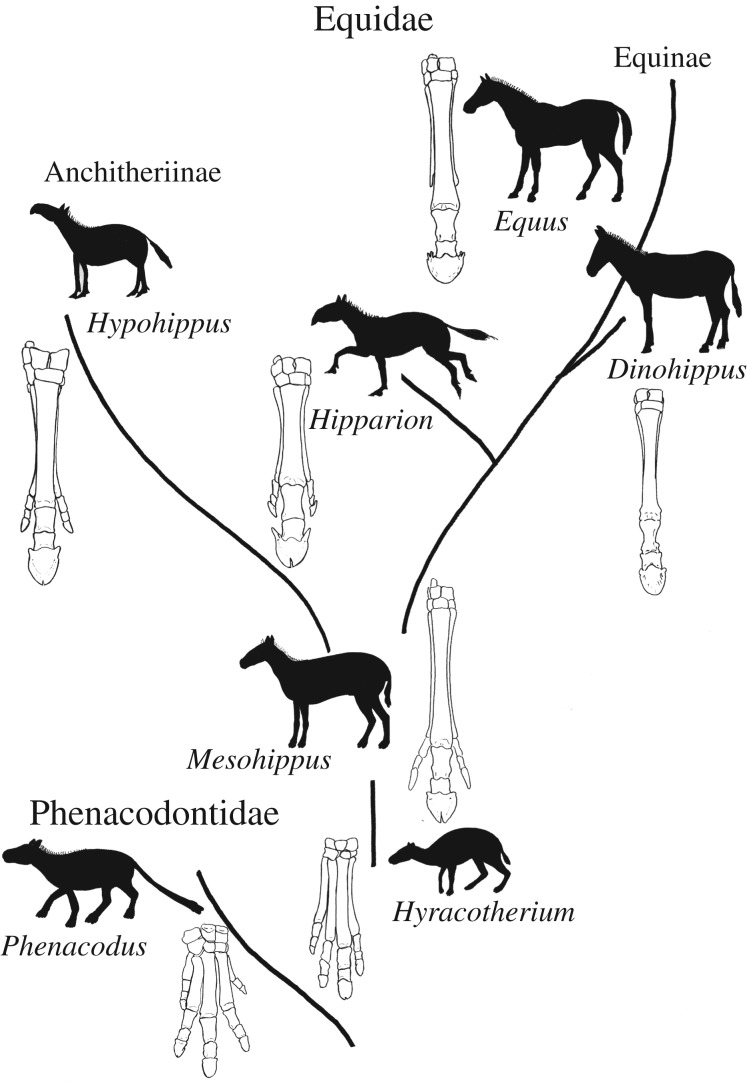
General evolutionary tree of the Equidae, and the changes in distal forelimb skeletal anatomy. *Phenacodus* is a primitive pentadactyl species that is close to the origin of the equid lineage. The equids commence as tetradactyl taxa during the Eocene, represented here by *Hyracotherium*. Later in the Eocene, the equids transition to tridactyly, with the oldest widespread taxon being *Mesohippus*. The equids split into two major subfamilies in the Early Oligocene: the Anchitheriinae and the Equidae. *Hypohippus* is a representative tridactyl anchithere from the Miocene, where the smaller side toes contact the ground. *Hipparion* represents the tridactyl equines of the Miocene; this species has shorter side toes that are suspended above the ground. The transition to monodactily begins in the Middle Miocene. *Dinohippus* is a monodactyl horse, known from the Plio-Pleistocene, and it marks the onset of the lineages that will become *Equus*. *Equus* is the only surviving genus of the Equidae. All the extinct equids demonstrated, aside from *Dinohippus*, have a median cleft on the terminal edge of the distal phalanx.

The evolution of equids has been studied extensively [5,11–13]. The basic pattern was an initial origin of *Hyracotherium* and other related archaic perissodactyl taxa from Condylarthra [14]. From *Hyracotherium* during the Eocene there are adaptive radiations (figure 1). As the taxa evolved, the forefoot (manus) changed from being tetradactyl to tridactyl and ultimately becoming monodactyl [7,14,15]. *Mesohippus,* a tridactyl horse, was one of the most common central taxa in the subsequent radiations. From *Mesohippus*, there were equids that evolved in two major directions. The Anchitheriinae were tridactyl taxa, had large lophed-like teeth and represented primarily browsers [13,16]. The majority of the Equinae were tridactyl and evolved dentitions that foreshadowed the modern horse. The teeth were more compact with complex infoldings of enamel and were hypsodont [6,12]. The hipparions were a subset of species within the Equinae that were common and widespread. They differed from *Equus* in having a tridactyl forefoot, having a deep pre-orbital fossa and in possessing an isolated protocone in the upper cheek teeth [17]. The Equinae evolved in the direction of grazers and ultimately gave rise to the modern horse [13,16].

This study includes common taxa that represent the major stages of horse limb evolution. We chose one pentadactyl taxon, *Phenacodus*, to represent the onset anatomy of the forefoot (manus). We selected a representative tetradactyl horse (*Hyracotherium*), tridactyl horse (*Mesohippus*) and monodactyl horse (*Dinohippus*) as the primary extinct horses for this study, as well as *Hipparion* to relate the limb anatomy to the only available fossil footprints from Laetoli.

### 3.3. Metacarpal osteology and anatomy of the phalanges

#### 3.3.1. Hyracotherium

**Figure 2.**
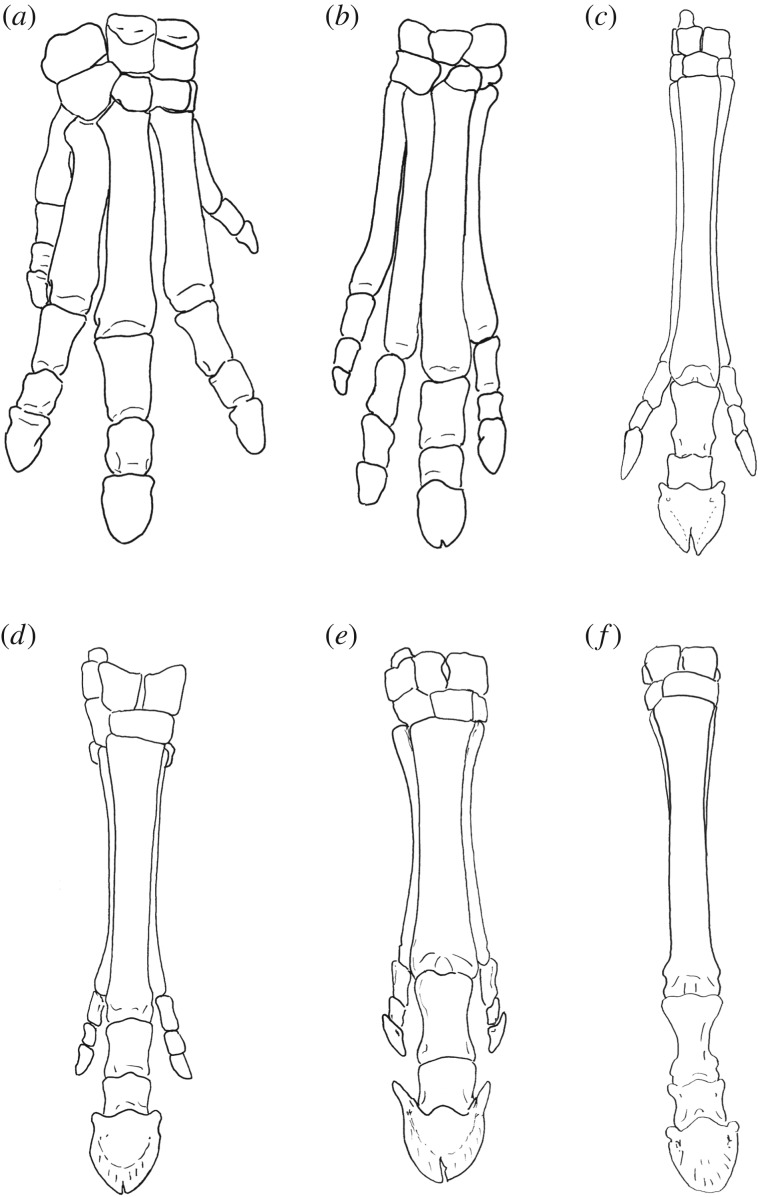
Distal forelimbs of the described extinct taxa in dorsal view. (*a*) *Phenacodus* (AMNH 4369), (*b*) *Hyracotherium* (AMNH 4832), (*c*) *Mesohippus* (AMNH 39480 and AMNH 1477), (*d*) *Hypohippus* (AMNH 9407)*,* (*e*) *Hipparion* (AMNH 109625), (*f*) *Dinohippus* (AMNH 17224). Each specimen is isometrically scaled so that all specimens are of equal length.

**Figure 3.**
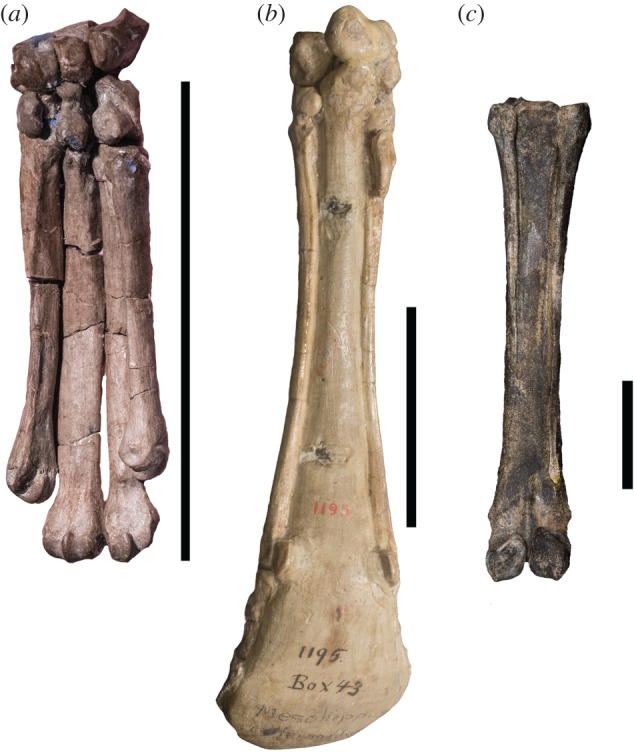
Metacarpal photographs of several extinct taxa in ventral view. (*a*) *Hyracotherium* (MCZ VPM-3440) and (*b*) *Mesohippus* (AMNH 1195). Note the presence of the small rudiment of metacarpal V on the ventral surface of metacarpal IV. (*c*) *Dinohippus* (AMNH 11635). Scale bars are all 50 mm.

The distal front limb consists of four complete digits. Metacarpal III is the widest and longest bone (figures 2*b* and 3*a*; table 1). Metacarpals II, IV and V are similar in shape, width and length, with metacarpal IV being slightly larger. On the ventral surface of metacarpal II, there is a small triangular expansion proximally that tapers distally onto a thin, faint ridge that extends onto the ventral shaft. There are no ridges on the ventral surfaces of metacarpals III–V when articulated; the surfaces of metacarpals III–V are slightly elevated where the metacarpals contact one another. The four metacarpals are approximated towards the wrist, and they splay outward distally towards the phalanges.

**Table 1.** Comparative anatomy of described extant and extinct equids.

	*Hyracotherium*	*Mesohippus*	*Dinohippus*	*Equus*
number of complete digits (metacarpal, proximal, middle and distal phalanges)	4	3	1	1
number of splint bones	0	0	2	2
presence of ridge on metacarpal II (or medial splint bone)	present	present	present	present
presence of ridge on metacarpal IV (or lateral splint bone)	absent	present	present	present
description of ridge	distinct, triangular raised textured area proximally, which tapers distally into a thin, raised ridge	textured region splaying outward proximally, tapering distally into a thin ridge with a fusion line on the inner surface of the ridge	textured surface that expands slightly outward proximally, extending distally into a ridge that gradually narrows, with a suture on the outer surface	textured, triangular, distinct region on the proximal edge with a textured, raised ridge extending distally down the shaft
shape of distal phalanx III	spade-shaped, with deep constriction proximally separating the angles from the wide splaying distal edge	narrow tubular shaped with pointed terminal edge, with a shallow constriction separating the angles from the wider distal edge	wedge shaped with crescenteric terminal edge	wedge shaped with crescenteric terminal edge that forms a ‘horseshoe' U-shape
presence (and depth) of median cleft on distal phalanx III	present (deep)	present (deep)	present (faint)	absent
presence (and size) of wings on distal phalanx III	absent	absent	present (large)	present (variable in size)
shape and orientation of wings of distal phalanx III	/	/	boxy shaped, flaring outward	triangular shaped, pointed dorsally

The phalanges of all four digits are similar in length and width, with III being slightly larger. The distal phalanges II, IV and V are tubular and elongated, with a notably arched dorsal surface. The distal phalanx of III is spade-shaped, with a deep constriction separating the angles from the distal edge, most visible in solar view. There is an elongated, distinct, wide median cleft on the terminal edge of the phalanges. The angles of the distal phalanges are notably blunted, and there are no wings.

#### 3.3.2. Mesohippus

The forelimb consists of three digits (II, III and IV), each with a complete metacarpal, proximal, middle and distal phalanx (figures 2*c* and 3*b*). Digit III is dominant; it is has the largest and widest metacarpal. The articular view at the wrist shows three distinct facets corresponding to metacarpals II, III and IV, and large rounded protrusions over digits II and IV. There is a textured region on the proximo-ventral surface of metacarpal II that splays outward and tapers distally into a thin ridge that extends down the shaft of the metacarpal. The ridge is distinct from the shaft of metacarpal II. Metacarpal IV is similar to metacarpal II in length and width. In some specimens, metacarpal V presents as a short, proximal, tubular protrusion on the ventral surface of metacarpal IV. In specimens where this rudiment is absent, there is a distinct ridge with a fusion line on the ventral surface of metacarpal IV, which extends almost completely down the shaft. The ridges on the surfaces of metacarpals II and occasionally of IV of *Mesohippus* resemble those of the modern horse.

The phalanges of the dominant digit are larger, fuller and wider than those of the side digits. The dominant distal phalanx is a single, elongated, tubular-shaped bone. In dorsal view, the bone is somewhat narrow and spade-shaped, with a deep median split on the terminal edge. There is a constriction in solar view separating the distal phalanx proper from the angles. In lateral view, the distal phalanx is shallow, and there are no medial or lateral wings above the angles. The side proximal and middle phalanges of digits II and IV are thin and complete. Laterally, each distal phalanx has a short blunted angle with no wing above it.

#### 3.3.3. Dinohippus

The metacarpals appear to be isometrically smaller than those of *Equus* (figures 2*f* and 3*c*). There is one dominant central digit III and two lateral splint bones (metacarpals II and IV). Metacarpals II and IV are full proximally and taper distally. On both metacarpals II and IV, there is a slightly expanded, textured area at the proximo-ventral area. This textured surface extends distally into a ridge at the ventral surface that gradually narrows. There is a faint depression on the outer surface of the ridge that separates the ridge from the remainder of the shaft. The distal aspect of metacarpals II and IV tapers into a thin rounded point.

There is one complete digit consisting of a proximal, middle and distal phalanx. The distal phalanx is a wedge-shaped bone that appears crescentic in dorsal and solar views. There is a small midline split on the terminal edge of the distal phalanx, which is most visible in dorsal view. The angles are present, but blunted. The wings are large, boxy and separated from the angles by a wide groove. The wings in dorsal view flare outward. The wings are separated into parietal and inner surfaces by a shallow groove.

#### 3.3.4. Equus

**Figure 4.**
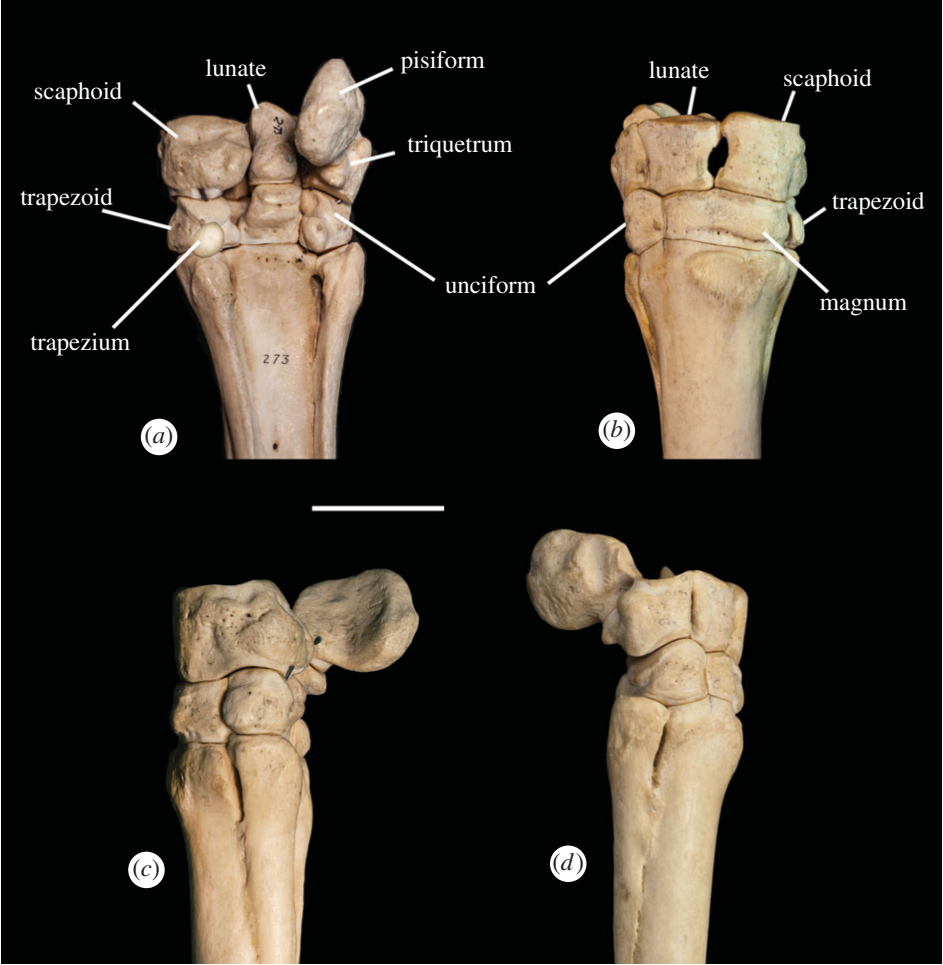
The *Equus* wrist (NS 273) in (*a*) ventral, (*b*) dorsal, (*c*) medial and (*d*) lateral views, showing the skeletal anatomy and carpal–metacarpal articulations. Scale bar is 50 mm.

**Figure 5.**
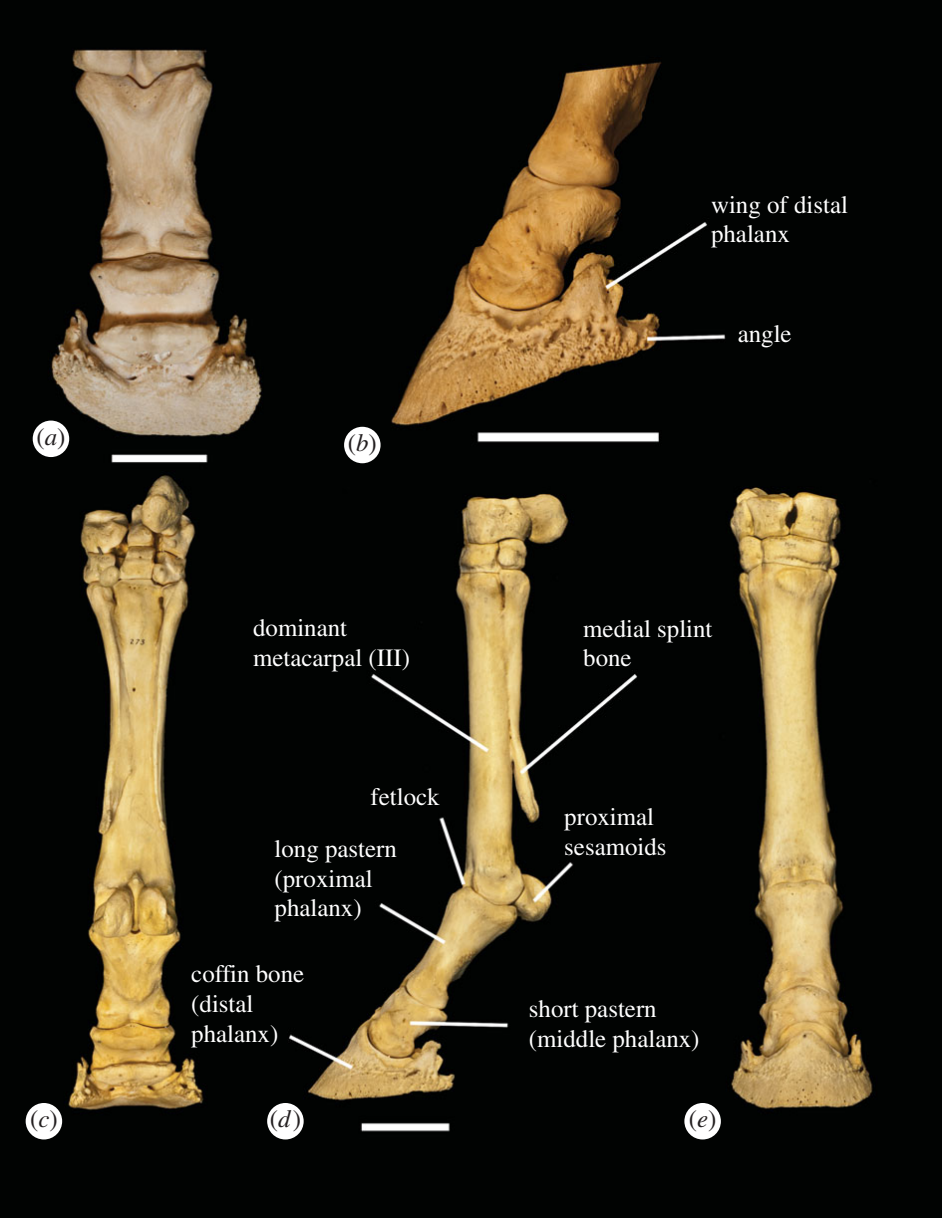
*Equus* distal forelimb skeletal anatomy. (*a*) Middle and distal phalanges in ventral view. (*b*) Middle and distal phalanges in medial view, depicting the angle and wing. (*c*) Distal forelimb in ventral view. (*d*) Distal forelimb in medial view. (*e*) Distal forelimb in dorsal view. Scale bars are all 50 mm.

The proximal row of carpals consists of four bones, and the distal row contains three to four bones, with the trapezium being variably present (figures 4 and 5). The metacarpal region consists of a dominant central bone (III) and two lateral bones (metacarpals II and IV), which are often partially or completely fused to the main digit. Metacarpal II has a triangular-shaped, elevated, textured region on the proximal edge that continues distally into a thin ridge that extends the majority of the length of the bone. The proximal area and ridge are textured and rough, and appear distinct from the smooth surface of the shaft in lateral view. Distally, metacarpal II flares outward and terminates in a large, flattened bulge. Metacarpal IV is rounded proximally. There is a large, elevated plateau with a textured surface at the proximal end of the bone, which extends distally down the shaft. Metacarpal II is smaller and thinner than metacarpal IV. Neither metacarpal II nor metacarpal IV ends in a synovial joint.

There are three phalanges on the functional manus: proximal (long pastern), middle (short pastern) and distal (coffin). The distal phalanx is single and wedge-shaped with a crescentic terminal edge that forms a broad symmetrical ‘U’ without a median split. In solar view, the crescentic surface terminates in two symmetrical pointed knobs, termed the angles, with one medial and the other lateral.

Above the angles there are two symmetrical dorsal protrusions named the wings, which are positioned medially and laterally. The wings are triangular/wedge-shaped and are pointed dorsally, paralleling the shape and orientation of the extensor tubercle. The size of the wings varies between individuals. The wings are separated into a parietal and inner surface by a deep groove, which houses a portion of the medial and lateral hoof cartilages. The hoof cartilages protrude behind the distal phalanx, and on the internal aspect, there are numerous ligaments.

### 3.4. Metacarpal–carpal and carpal–carpal articulations

#### 3.4.1. Phenacodus

**Figure 6.**
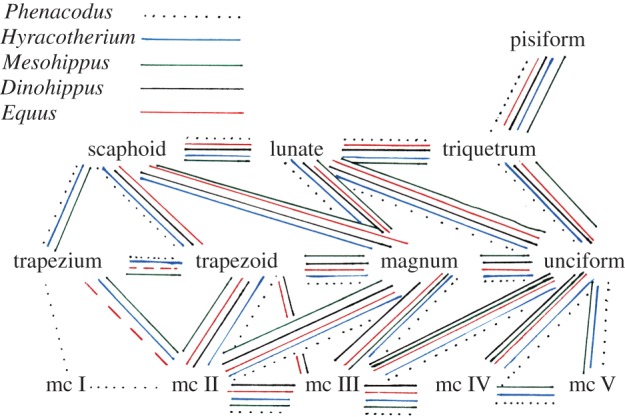
Schematic of the metacarpal–carpal and carpal–carpal articulations. Dotted black lines represent *Phenacodus* articulations. Blue lines represent *Hyracotherium* articulations. Green lines represent *Mesohippus* articulations. Black lines represent *Dinohippus* articulations. Red lines represent *Equus* articulations (dashed red lines indicate articulation is variable).

Metacarpal–carpal: Metacarpal I articulates laterally with metacarpal II (one small facet) and proximally with the trapezium (one large facet) (figures 2*a* and 6). Metacarpal II articulates laterally with metacarpal III (one small facet), and proximally with the trapezoid (one large facet) and magnum (one small facet). Metacarpal III articulates laterally with metacarpal IV, and proximally with the magnum (one large facet) and unciform (one small facet). Metacarpal IV articulates laterally with metacarpal V (one small facet) and proximally with the unciform (one large facet). Metacarpal V articulates proximally with the unciform (one large facet).

Carpal–carpal: The trapezium articulates dorsally with the trapezoid (one small facet) and proximally with the scaphoid (one small facet). The trapezoid articulates laterally with the magnum (two small facets) and proximally with the scaphoid (one large facet). The magnum articulates laterally with the unciform (one large facet) and proximally with the lunate (one large facet). The unciform articulates proximally with the lunate (one small facet) and triquetrum (one large facet). The scaphoid articulates laterally with the lunate (two small facets). The lunate articulates laterally with the triquetrum (two small facets). The triquetrum articulates ventrally with the pisiform (one large facet).

#### 3.4.2. Hyracotherium

Metacarpal–carpal: The same articulations as in *Phenacodus* are present, aside from the articulations of metacarpal I, as this digit is absent in *Hyracotherium* (figures 2*b* and 6). In *Hyracotherium*, there is an articulation between metacarpal II and the trapezium.

Carpal–carpal: The same articulations as in *Phenacodus* are present, with the addition of an articulation between the magnum and the scaphoid.

#### 3.4.3. Mesohippus

Metacarpal–carpal, carpal–carpal: The same articulations as in *Hyracotherium* are noted (figures 2*c* and 6).

#### 3.4.4. Dinohippus

Metacarpal–carpal: Metacarpal II articulates laterally with metacarpal III (two small facets), and proximally with trapezoid (one large facet) and magnum (one small facet) (figures 2*f* and 6). Metacarpal III articulates laterally with metacarpal IV (two small facets), and proximally with the trapezoid (one small facet), magnum (one large facet) and unciform (one small facet). Metacarpal IV articulates proximally with the unciform (one large facet).

Carpal–carpal: The same articulations as in *Hyracotherium* and *Mesohippus* are noted, aside from the apparent lack of trapezium in the examined *Dinohippus* specimens.

#### 3.4.5. Equus

Metacarpal–carpal: The same articulations as in *Dinohippus* are present, with the addition of an articulation between metacarpal II and the trapezium, when present (figures 4 and 6).

Carpal–carpal: The same articulations as in *Hyracotherium, Mesohippus* and *Dinohippus* are noted.

### 3.5. Metacarpal morphology and articulations of *Hypohippus*

*Articulations*:

Metacarpal–carpal: The same articulations are present as in *Phenacodus*.

Carpal–carpal: Mostly the same articulations are present as in *Phenacodus*, with the addition of an articulation between the magnum and scaphoid.

There is one dominant metacarpal (III) and two side digits (II and IV), which are complete with a metacarpal, as well as with proximal, middle and distal phalanges (figure 2*d*). Metacarpal III is wider and slightly longer than the side metacarpals, which are thin, elongated and symmetrical. Rudiments of both metacarpals I and V are seen at the proximal shafts. These rudiments are approximately 1 cm long and are positioned on the ventral surfaces of metacarpals II and IV, respectively.

### 3.6. The frog and keratinous hoof of *Equus*

#### 3.6.1. Frog

**Figure 7.**
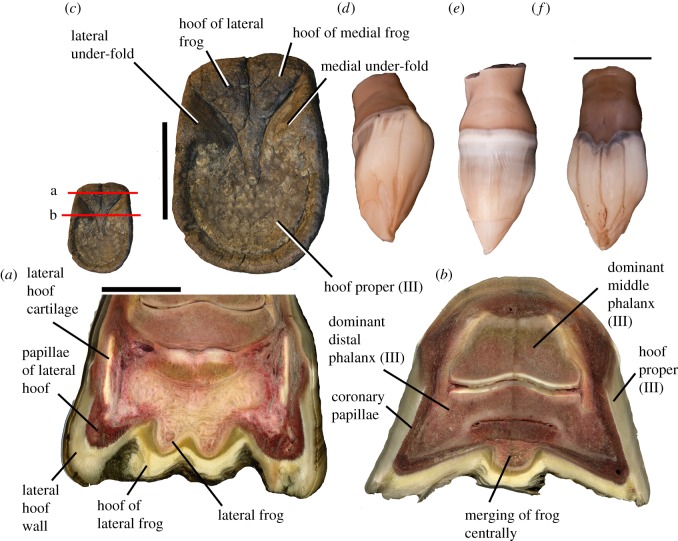
The frog and keratinous hoof of *Equus*. (*a*) Cross section of adult *Equus* hoof (NS 297). The frog is a double-sided connective tissue structure. The frog has its own hoof, which is softer than the hoof proper; the lamellar fibres here are distinct from and perpendicular to the fibres of the lateral and medial sides of the hoof. Directly ventral to the hoof cartilages, there are distinct papillae and lamellar fibres. (*b*) Cross section of the hoof distally, where the frog has merged at the centre. The fibres of the hoof of the frog are also merged, but are still distinct from the hoof proper. There are two keratinous under-folds each with a distinct orientation of fibres. (*c*) Ventral view of keratinous hoof, showing the V-shaped frog and keratinous under-folds. The entire distal area (towards the crescentic terminal edge) is uniform and represents the hoof proper (III). (*d*) Fetal hoof (NS 292) in lateral view. (*e*) Hoof in dorsal view, with a smooth, undivided surface. (*f*) Hoof in ventral view, with four distinct infoldings. Scale bar for (*a–c*) is 35 mm and (*d–f*) is 20 mm.

**Figure 8.**
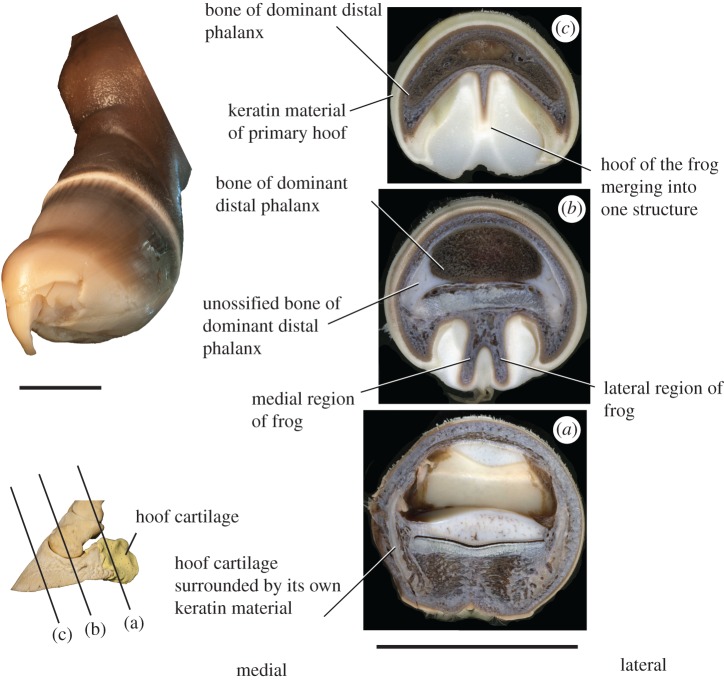
Gross and internal anatomy of a full-term *Equus* fetal hoof (NS 296). (*a*) Proximal-most section, where the frog is a large, double-sided structure. On either side of the section, there is a hoof cartilage surrounded by keratin distinct from the hoof proper. (*b*) Middle section through the hoof, in the region where the frog presents as two distinct ridges ventral to the distal phalanx. (*c*) Distal-most section, where the frog is merged centrally into a single structure. There is keratinous material surrounding the distal phalanx, comprising the hoof proper. (*d*) Fetal hoof before sectioning, showing a curved, keratinous claw at the terminal edge. Scale bar for (*a–c*) is 50 mm and for (*d*) is 20 mm.

The frog is a dense fibrous structure that contacts the ground (figures 7*a,b* and 8). It is situated between the medial and lateral hoof cartilages on the ventral surface of the distal phalanx. Ventral view of the frog reveals a single median structure proximally, which separates into two bilateral symmetrical ridges distally, forming a V. The two symmetrical ridges are clearly distinct from the main distal phalanx. The frog is surrounded by soft keratinous lamellae, which is a type of hoof. The frog is capped by keratinous lamellae with fibres perpendicular to and discontinuous with the fibres of the hoof proper. The lamellae of the frog are oriented horizontal to the ground. The frog has distinct corium and papillae with germinating properties [18].

#### 3.6.2. Keratinous hoof

There are several parts to the keratinous horse hoof (figure 7*a–c*). The dorsal surface of the hoof has a keratin-generating region proximally. The numerous layers of keratinous lamellae grow distally over the dorsal surface of the distal phalanx. Between the dorsal surface of the distal phalanx and the hoof keratin is the nail bed (corium of the wall), which also has germinating properties. This structure adheres to the dorsal periosteum of the distal phalanx. There are additional generating regions on the ventral surface of the hoof, forming ventral lamellae. The white line (a tubular horn between the lamellae) is formed by the junction of the dorsal lamellae and the ventral lamellae. In the human nail, the nail bed terminates at the apex [19], whereas in *Equus*, there is additional hoof (nail) material extending ventral to the apex, growing from a nail bed on the ventral distal phalanx as well as on the frog. There are under-folds of keratin at the lateral and medial aspects of the proximal hoof, which are distinct from the frog and the sole of the hoof proper. These under-folds are positioned in the region ventral to the medial and lateral hoof cartilages. On the ventral surface of the hoof, there are five regions, each with their own orientation of lamellar fibres; two sections are under the frog, two are at the medial and lateral under-folds, and the fifth and largest region comprises the remaining hoof surface towards the crescentic edge.

The hooves of fetal specimens are softer than those of adult individuals. Externally, our horse fetus exhibits four distinct infoldings on the ventral region hoof, whereas the dorsal region of the hoof is smooth without subdivisions. These folds are distinct distally and blend together proximally towards the middle phalanx (figure 7*d–f*).

### 3.7. *Hipparion* footprints from Laetoli

**Figure 9.**
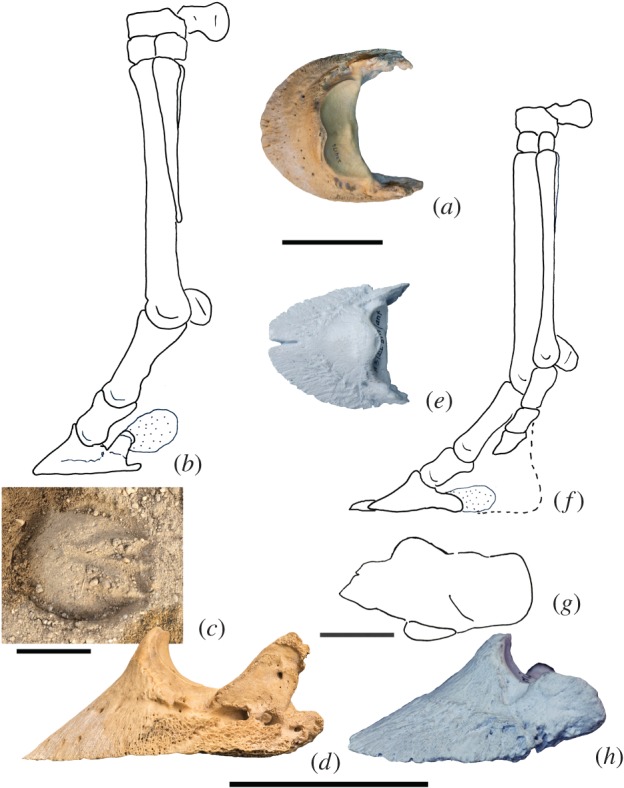
The distal forelimbs of *Equus* and *Hipparion* with footprints. (*a*) *Equus* distal phalanx (AMNH 2014176) in dorsal view, showing the absence of the median cleft. (*b*) Distal forelimb of *Equus* in medial view, showing the skeletal anatomy and medial wing. (*c*) Footprint of the modern horse, showing an uninterrupted arc anteriorly (classic horseshoe shape), and V-shaped impression posteriorly, created by the frog. (*d*) *Equus* distal phalanx in medial view, with a large wing. (*e*) *Hipparion* distal phalanx (AMNH 109625) in dorsal view, with a notable median cleft. (*f*) Distal forelimb of *Hipparion* in medial view, demonstrating the large wing of the distal phalanx. The side toes are complete and are notably shorter than the dominant digit III. Dotted line indicates a proposed paw. (*g*) Outline of the famous *Hipparion* footprint from Laetoli, Tanzania. (*h*) *Hipparion* distal phalanx in medial view, with a large wing. All scale bars are 60 mm.

There is a set of well-known footprints at Laetoli in Tanzania (approx. 4 Ma, Pliocene), including the tracks of *Hipparion* [20]. These tracks are notably different from those of a modern horse. In the footprint of *Equus*, there is a single impression that is rounded anteriorly, with a V-shaped indentation at the posterior aspect. In the tracks of *Hipparion*, the anterior-most aspect of the print terminates in a sharp median point, there is no V-shaped impression posteriorly, and there are two additional side impressions slightly posterior to the dominant impression. In addition, they also show a large round impression behind the hoof (figure 9).

The *Hipparion* forefoot was tridactyl, with digit III being dominant, and digits II and IV as the fully developed side limbs (figure 2*e*). The forelimb anatomy closely parallels that of the other tridactyl equids, including our previously described *Mesohippus* limbs. The *Hipparion* distal phalanx exhibits well-developed wings above the angle. These wings are oriented horizontally and are positioned close to the solar surface of the distal phalanx, while the angles are blunted. There is a deep cleft at the median plane of the terminal edge of the distal phalanx. The side digits are thinner and shorter than the dominant digit. The side distal phalanges lack the wings seen in the dominant distal phalanx, but possess a pronounced single angle on the outer edge of the hoof.

### 3.8. Nerve and vessel distribution in *Equus*

**Figure 10.**
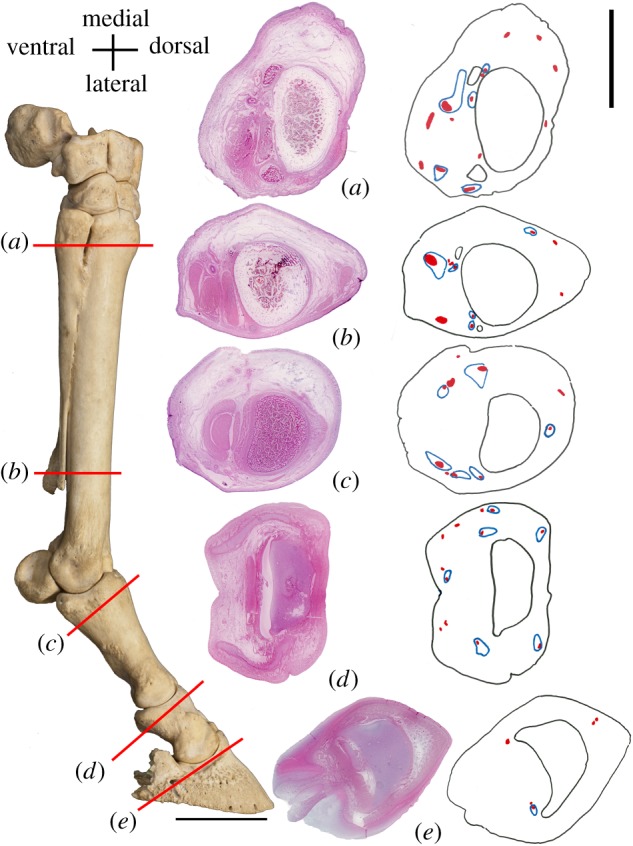
Nerve and vessel distribution in *Equus* histological specimens. Specimen and bones are outlined in black, nerves are outlined in red and triads of vein, artery and nerve (VAN) are outlined in blue. (*a–e*) Histological slides (40× magnification) of the *Equus* manus (NS 290) in transverse sections, with a drawn distribution of the large nerves and VAN triads for each section. Scale bar for histological slides is 14 mm and for skeletal forelimb is 60 mm.

Fourteen distinct nerve bundles characterize the proximal end of the horse manus. We find two to four nerve bundles positioned dorsal to metacarpal III, one nerve bundle adjacent to the inner surface of each of metacarpals II and IV, and one to two nerves adjacent to the outer surface of metacarpals II and IV. In addition, there are three to four large nerve bundles located ventrally in the proximity of the muscle groups, and an additional small nerve bundle is situated in the region ventral metacarpal III. In this area, there are two to three distinct bundles of associated vein, artery and nerve (VAN) around metacarpal II and two VANs around metacarpal IV (figure 10*a*)

Approximately three-quarters of the way down the metacarpal, 8 to 10 nerves are found. Two to three nerves are positioned dorsal to metacarpal III, and the remaining nerves are positioned ventrally. There are two large nerve bundles ventrally, one lateral and one medial. Three smaller nerve bundles are seen adjacent to the distal end metacarpal II and two bundles are found adjacent to the distal end of metacarpal IV. We find five distinct VANs; one to two large VANs ventrally (one medial and one lateral), three smaller VANs in the ventral region and zero to one VANs dorsally (figure 10*b*).

At the cross section through the proximal phalanx, 9 to 10 nerves are seen, with one to two nerves positioned dorsally to the proximal phalanx. There are two large nerve bundles on the ventral region, one lateral and one medial. There are four intermediate-sized nerve bundles, with two positioned laterally and two medially, which are relatively symmetrical in position. We find two additional small nerve bundles ventrally, one lateral and one medial. We find five to six VANs total in this region. We observe two large VANs (one medial and one lateral). There are one to two additional smaller VANs laterally, one small VAN medially and one VAN dorsally (figure 10*c*).

A transverse section through the middle bony phalanx reveals 9 to 12 nerves, which are overall smaller than the proximal counterparts. We find one to three small nerve bundles positioned dorsally to the middle phalanx, and the remaining nerve bundles are positioned ventrally. In the ventral region, there are two, symmetrical, intermediate-sized nerve bundles, one lateral and one medial. There are several nerves surrounding the lateral hoof cartilage. We find two large VANs ventrally (one medial and one lateral), and four to five additional smaller VANs surrounding the middle phalanx (figure 10*d*).

At the most distal section, five to six nerves are identified, with two to four nerves surrounding the dorsal aspect of the distal phalanx. We find two to three additional nerve bundles are positioned just ventral to the distal phalanx, one to two laterally and one medially. We find a single VAN triad ventral to the distal phalanx, and numerous minute arteries throughout the section. There is a reduction in large nerve number from the proximal counterparts. In this region, the nerves are numerous, but the size is notably reduced, possibly reflecting significant branching of the more proximal nerve segments (figure 10*e*).

## 4. Discussion

Limb reduction has occurred several times during tetrapod evolution [21]. This can occur during mammalian development by reducing the number of digit precursors during embryogenesis, where even the earliest embryos commence with fewer than five digits, or by forming an initially pentadactyl limb and subsequently removing digits via apoptosis and degeneration [22]. Limb reduction as a result of a spontaneous gene mutation is often associated with several negative pleiotropic effects [23]. This is due to the timing of limb signalling during the phylotypic stage, where several important embryonic events occur, including neurulation and somite formation [24]. However, if this digit reduction occurs over a long evolutionary process, such as millions of years as it occurs in the equid lineages, the evolutionary constraint is decreased [25].

Tetrapod forelimbs are organized into the stylopod (humerus), zeugopod (radius and ulna) and autopod segments (carpals, metacarpals and phalanges) [26]. During limb evolution, the development of the distal autopod components occurs later than the more proximal stylopod and zeugopod segments [27]. Interestingly, the distal limb elements have a greater degree of morphological and structural variation than the more proximal counterparts, possibly due to their more recent evolution [28,29]. Correspondingly, we find that the horse distal limb elements are variable throughout equid evolution, whereas the proximal segments of the limb are more conservative.

**Figure 11.**
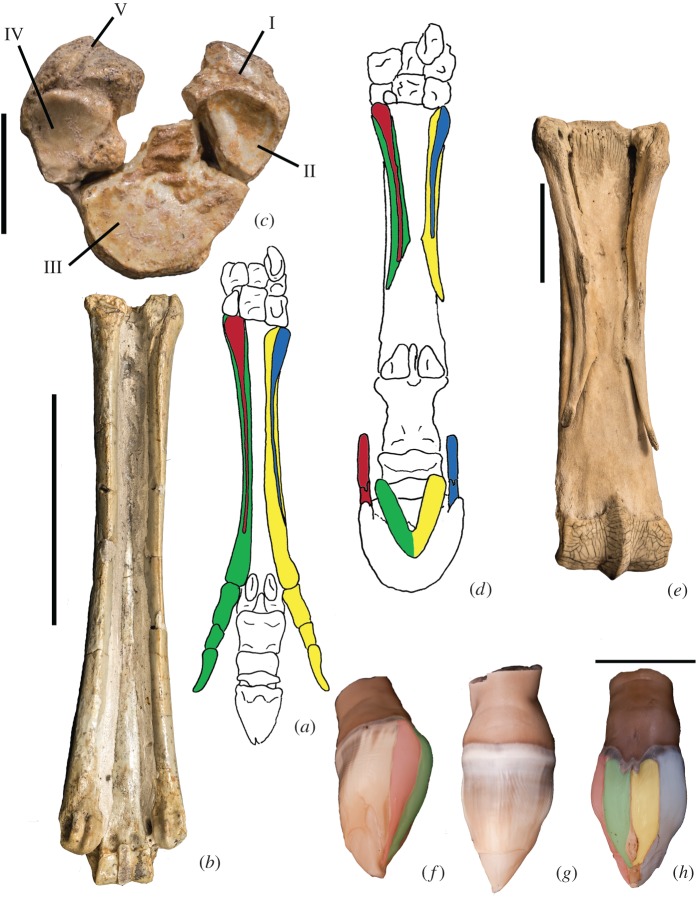
Proposed positioning of the five digits in the distal forelimb of *Equus* and *Mesohippus*. Digit I is in red, digit II is in green, digit III is dominant and uncoloured, digit IV is in yellow and digit V is in blue. (*a*) Ventral view of the *Mesohippus* forelimb. The ventral ridges on the side metacarpals represent reduced metacarpals I and V, consistent with their locations in the modern horse. (*b*) Ridges on the splint metacarpals in *Mesohippus* (AMNH 39480)*.* Scale bar is 50 mm. (*c*) Articular view of the metacarpals of *Mesohippus.* Scale bar is 10 mm. (*d*) Ventral view of *Equus* manus. The medial splint bone represents the reduced metacarpals of I and II, and the lateral splint bone represents the reduced metacarpals of IV and V, with I and V positioned on the ventral splint bone shafts. At the distal phalanx, the bony wings and associated hoof cartilages represent the distal digits I (medially) and V (laterally). Ventrally on the hoof, the frog represents the partially differentiated distal digits II and IV. (*e*) Ridges on the splint metacarpals in *Equus* (AMNH 204183). Scale bar is 50 mm. (*f*) Medial view of fetal horse hoof. (*g*) Dorsal view of the fetal hoof, showing a smooth singular surface, representing the dominant digit III. (*h*) Ventral view of a fetal horse specimen, showing four distinct infoldings that depict our proposed digits I, II, IV and V. Scale bar for (*f–h*) is 20 mm.

Using the osteology of the modern horse when compared to more primitive taxa with less substantial limb reduction as well as the nerve and arterial distribution, wrist articulations, and anatomy of the frog and keratinous hoof, we find evidence to support that *Equus* retains the identities of digits I–V, most notably at the wrist and distal hoof (figure 11). Consistent with homologies in the literature, we believe that the dominant metacarpal represents metacarpal III, and the splint bones are reduced metacarpals II and IV. We propose that metacarpals I and V are expressed as ridges on the ventral surfaces of metacarpals II and IV. Distally on the hoof, we theorize the medial and lateral hoof wings and cartilages represent the distal aspects of digits I (medial) and V (lateral), and that the dominant digit including the proximal, middle and distal phalanges, represents digit III. Lastly, we propose that the frog portrays the distal aspects of digits II and IV. The reduction in the horse resembles an hourglass, where all digits are expressed proximally and distally; however, in the middle portion (distal metacarpal, proximal and middle phalanges), only digit III is prominent.

The osteology of the *Equus* manus supports our theory that all five digits, rather than the traditionally conceived three digits, are expressed proximally. *Equus* is commonly described as a monodactyl taxon, with digit III representing the main and only complete digit and metacarpals II and IV reduced into the medial and lateral splint bones respectively. We propose that metacarpals I and V are also expressed on the surfaces of metacarpals II and IV. The proximo-ventral surfaces of metacarpals II and IV are textured with distinct ridges extending down the shafts that are distinct from the remaining smooth surfaces. We believe these surface irregularities represent digits I and V.

Side metacarpals II and IV have a ridge on the ventral surface, which is a shared feature of the extinct monodactyl *Dinohippus*, tridactyl *Mesohippus* and *Equus*. We propose these textured bony elevations, which are distinct from the smooth splint metacarpal shafts, represent surface expressions of metacarpals I and V, digits previously believed to be lost in these taxa (figure 11). In *Mesohippus*, the line of fusion between metacarpals IV and V is visible at the inner surface of the ridge (figure 11*b*), and in *Dinohippus*, a faint suture line is visible at the outer surface. We believe these fusion lines indicate that the ridges are separate structures from the splint metacarpals, digits I and V, that throughout time have embedded onto the surfaces of digits II and V. In *Hyracotherium,* where digits II–V are fully present, only metacarpal II possesses a clear ventral ridge. This resembles the elevation seen on metacarpals II and IV in *Equus*, where it is expanded and triangular proximally and tapers distally into a thin ridge. The lack of ridges on the other metacarpals of *Hyracotherium* reinforces this hypothesis, as all other digits are already present. Metacarpals III–V of *Hyracotherium* are, therefore, singular and do not contain remnants of other digits, as is seen in later equids.

This proposed location of metacarpals I and V is consistent with the extinct tridactyl *Hypohippus*, where all five digits are present proximally towards the wrist. In *Hypohippus*, metacarpals I and V are present at the proximal shaft, as ovoid bony elements positioned on the ventral surface of metacarpals II and IV. These rudiments of the incomplete metacarpals I and V are in the same position as the ventral surface irregularities in *Equus*, *Dinohippus* and *Mesohippus.* This further supports the identities of the ventral ridges and textured regions in the monodactyl and tridactyl horses as lost digits I and V. The digit identities of the fragments as I and V in *Hypohippus* are confirmed by carpal articulations; the rudiment of I articulates with the trapezium, and the rudiment of V articulates with the unciform.

Interestingly, despite a reduction in the number of complete digits and an increase in their size, the overall positions of individual elements and their borders in the manus has remained consistent throughout equid evolution. We see evidence of this evolutionary history in the anatomy of the modern horse forelimb. The horse carpus and manus retains the same borders of individual bones that were seen throughout equid lineages. Moreover, the orientation and general structure of the *Equus* hand reflects the evolutionary history. In the tetradactyl *Hyracotherium*, the four complete digits are not aligned horizontally, but instead curve inward, creating a tubular structure. This orientation remains consistent in the tridactyl *Mesohippus* manus, where side digits II and IV arch inward in relation to dominant digit III, and the remnants of digits I and V are positioned ventral to the side digits. In the modern horse, the manus maintains this overall curved orientation created by splint digits II and IV and their ventral ridges representing reduced digits I and V. Therefore, the transition from the tetradactyl equids to the modern horse was not as dramatic as previously thought.

The trapezium of *Equus* is small and variably present [30,31], whereas in the extinct equids, it is large and almost always present. Owing to the reduction of the trapezium, it loses its articulation proximally with the scaphoid, but maintains its articulation ventrally with the trapezoid and occasionally distally with the ventral aspect of metacarpal II (proposed metacarpal I). The presence of the trapezium in the horse, and the occasional distal articulation suggests that digit I is not completely missing, as is previously suggested in the literature. Ruminants lack skeletal evidence for digit I, and correspondingly the trapezium is absent [32–34]. We believe the presence of the trapezium, combined with the surface irregularity of metacarpal II indicates the presence of metacarpal I in the horse.

Using our proposed identities of the five digits in the *Equus* manus, the carpal–metacarpal articulations are consistent with those in *Phenacodus*, a primitive pentadactyl taxon (figures 2*a* and 4). The bony articulations in the horse remain consistent throughout evolution despite apparent digit reduction, and retain relations to their traditional borders. In *Phenacodus*, metacarpals I and II articulate with the trapezium and trapezoid, respectively. In *Equus*, the medial splint bone (proposed metacarpals I and II) articulates with the trapezoid and occasionally ventrally with the trapezium. In *Phenacodus*, metacarpals IV and V articulate with the unciform, which is consistent with our proposal that the lateral splint bone, which articulates with the unciform, represents metacarpals IV and V. The metacarpal–carpal relations have remained the same regardless of apparent digit reduction in the horse, supported by the similarities in articulations between the ‘monodactyl’ *Equus* and the pentadactyl *Phenacodus* distal forelimbs.

Among the extinct equids described, only *Hyracotherium* has a difference in the number of digits in the forelimb versus the hindlimb. In *Dinohippus* and *Equus*, both the forelimb and hindlimb are monodactyl, and in *Mesohippus*, the front and back limbs both consist of three complete digits. In *Hyracotherium*, the forelimb is tetradactyl and the hindlimb is tridactyl. Interestingly, the *Hyracotherium* foot possesses small splint digits on the ventral surfaces of metatarsals II and IV, which represent ‘missing’ metatarsals I and V [35,36]. The position of these metatarsal rudiments in the *Hyracotherium* hindlimb foreshadows the rudiments in the hands of the later equids; *Mesohippus* and *Hypohippus* possess elements of metacarpals I and V on the ventral surfaces of complete digits II and IV, respectively. Unlike the limbs of *Hyracotherium*, the forelimbs and hindlimbs of the other described equids are notably similar, both in number of digits and overall morphology.

We also find morphological evidence for partially developed digits I, II, IV and V distally on the phalanx and hoof of *Equus*. The distal bony phalanx of the hoof is digit III. On the medial and lateral sides of the distal phalanx, there are firmly attached small bony thickenings termed the wings, which resemble small distal phalanges but appear to be fused with the bony distal phalanx proper. We propose that the wings and hoof cartilages represent the distal aspects of digits I medially and V laterally. These wings are present in the tridactyl taxon *Hipparion*, where digits II–IV are fully formed, and are absent in the tetradactyl *Hyracotherium*, which only lacks digit I. We expected to find a medial wing on the distal phalanx of *Hyracotherium*, representing the single missing digit, but this taxon shows no apparent evidence of the pollex distally. We suggest that the presence of these ‘miniature hooves’ in *Equus* and *Hipparion* indicates that digits I and V are partially expressed distally, and are not absent as previously suggested. Correspondingly, on the hoof region directly ventral to the cartilages, there are distinct areas each with their own corium and keratinous lamellar fibres, which we propose represent the hooves of digits I (medially) and V (laterally).

The metacarpals of digits II and IV are located ventral to metacarpal III. If these digits were to continue distally, their projected position would be ventral to the dominant distal phalanx, consistent with the location of the double-sided frog. Embryonic studies of the distal horse hoof demonstrated regions of apoptosis surrounding the precursors for digits II and IV [22]. We find, however, morphological features indicating that II and IV, although largely reduced, still persist as incomplete digits. The frog is newly theorized to represent a partial differentiation of the distal parts of digits II and IV. These are the complete side digits in *Mesohippus* and the other tridactyl horses. The frog is absent from other living perissodactyls, including the tapir and rhino, as these taxa both have digits II and IV fully expressed [37]. Internally, the frog of *Equus* contains fibrous material (cushion) surrounded by keratinous lamellae that are distinct from the fibres of the hoof proper. The frog has its own germinal matrix, corium and papillae [18]. We propose the two distal segments of the frog are the partially differentiated digits II and IV, explaining the presence of hoof material and the germinating matrix that are distinct from the hoof proper.

On the ventral surface of the hoof, there are five distinct regions, each with their own orientation of lamellar fibres. The five regions on the ventral surface of the hoof represent the partially differentiated hooves of all five digits. The medial and lateral under-folds are positioned directly ventral to the medial and lateral hoof cartilages (proposed digits I and V), respectively. These under-folds represent the hooves of these digits. Centrally, there are two additional keratin regions with distinct fibre orientation positioned directly under the frog; these represent the hooves of digits II and IV. The remainder of the ventral hoof is singular and without subdivisions, and is fused to the solar surface of distal phalanx III. This represents the hoof of the main digit III. These five regions each have a distinct orientation of lamellar fibres from each other and separate areas of corium and papillae. The positions of these hoof subdivisions are consistent with our proposed position of digits I, II, IV and V both proximally and distally, where these digits are compressed onto the ventral aspect of the dominant digit.

In late-term fetuses, the keratinous hooves resemble an unopened tulip flower (figure 7*d–f*). The common number of ‘petals’ is four on the ventral side and one on the dorsal. The external appearance of the hoof parallels our observation of the adult hoof subdivisions. Ventrally, the four folds of hoof material represent the compressed digits I, II, IV and V, and the single dorsal region represents the hoof of digit III. The ‘lost’ digits are visible in the horse embryo as they are in the adult individuals, and again these digits are positioned ventral to dominant digit III. Most distally these five petals, although they externally display distal borders, merge into a single undifferentiated cone, which extends further and terminates into a notably long and curved point like a median claw. In the literature, these have been termed ‘soft cellular mass’ [38]. As the newborn walks these terminal structures slough off. We believe that if the horse retained this claw into adulthood, the distal phalanx would contain the midline cleft that characterizes most extinct equid taxa.

We are proposing that all five digits are present proximally and distally on the horse and ancestors, and that the middle portion of the shaft is absent. This hourglass pattern of reduction where the mid-portion is lost also occurs in fibular reduction in certain artiodactyls. In Pecora, the proximal fibula is present and fused to the lateral tibial condyle, and the distal fibula is represented in the malleolus. In these taxa, the middle portion of the fibula is completely reduced [39]. The bat fibula is reduced similarly, where the proximal-most head and the distal shaft are present; however, the proximal shaft is lost, therefore creating an interruption in the bone [40]. The ulna of several groups also follows this pattern of reduction where the central portion of the bone is less developed. In bats, camelids, cervids, antilocaprids and bovids, the proximal and distal regions of the ulna are well developed, whereas the middle of the shaft is notably reduced in width [39]. Our proposed reduction of the horse digits follows this hourglass pattern of reduction; the proximal digits are retained as the splint metacarpals and their ventral ridges, and the distal digits are retained as the frog and wings of the distal phalanx; however, the central portion of the digits is lost.

The Laetoli footprints of *Hipparion* sp. indicate a large difference in the distal limb anatomy from *Equus*. The footprints of this taxon were interpreted to indicate a ‘running walk’ gait, different from the modern horse [41]. A typical horse footprint includes an uninterrupted U-shaped arc anteriorly and inverted ‘V’ posteriorly, created by the frog. In the *Hipparion* impressions, the anterior aspect is pointed at the midline and there are two smaller side impressions medial and lateral to the main print. Studies have suggested that the side impressions represent the complete splint digits (II and IV) [5,41,42]. However, the side digits of *Hipparion* are shorter than digit III [43], and physical constraints might have prevented the side hooves from contacting the mud to create the impression [6]. We propose an alternative interpretation of the Laetoli footprints, where the side impressions represent expansions of the wings of the distal phalanx (our proposed digits I and V). The wings of *Hipparion* skeletal specimens are positioned just above the solar surface of the dominant distal phalanx, and could plausibly leave impressions in the mud if enveloped in their own keratinous hoof material.

The Laetoli *Hipparion* footprints lack the V-shaped posterior impression that characterizes the footprint of the modern horse. This posterior impression in the horse is created by the frog, which we propose represents distal digits II and IV. The *Hipparion* print lacks this V-shaped posterior impression, indicating the absence of a frog in this taxon. In *Hipparion*, digits II and IV are fully formed, and therefore, it should not have a frog. In place of the position of the frog, the *Hipparion* footprints exhibit a large round impression, which was hypothesized as mud stuck behind the hooves [20]. We propose that this impression was created by a posterior paw, similar to the limbs of living perissodactyls (rhinos and tapirs) [37].

We interpret the anterior point in the Laetoli footprints as the presence of a midline claw in *Hipparion*. The distal phalanges in hipparions and most other extinct equids exhibit a distinct median cleft on the terminal edge, creating an interruption in the U-shaped arc. A keratinous claw fused to the main hoof would explain the presence of this cleft. Such a cleft is present throughout most Equinae and Anchitheriinae. The midline cleft on the distal hoof is absent in adult horse specimens. We observe, however, the presence of the claw in fetal specimens of *Equus*, which is worn off before horses reach adulthood.

Just as the skeletal and hoof anatomy shows hints of the ‘missing digits’, the nerves of the horse show a pattern more similar to the pentadactyl forefoot anatomy, and are not reduced enough to reflect a single digit. At cross-sectional view through the five metacarpals, humans possess approximately 9 to 15 nerves [44,45]; the equivalent region in the horse shows 14 nerves (figure 10). Distally on each human finger, there are two nerves representing the proper palmar digital nerves, while the horse has 8 to 10 nerves at the phalangeal regions, reflecting the nerves of five digits combined. The arteries of the horse limb are also more numerous than expected for a monodactyl taxon. We acknowledge that the neurovascular supply may reflect the function of the limb and not solely the number of digits present.

The human finger exhibits two large triads of vein, artery and nerve (VAN) per finger, each consisting of a proper palmar digital artery, proper palmar digital nerve and associated vein [46]. These triads run down the medial and lateral ventral surface of the finger, providing the primary blood supply and innervation to the digit. In the horse cross sections, we find between five and seven distinct triads in the phalangeal region; this is greater than the two VANs that would be expected if the horse limb truly consisted of one digit, but less than the 10 total VANs expected for five combined digits. We propose that five digits are partially present but are not fully differentiated, and therefore, the vasculature reflects a state intermediate between a true monodactyl and true pentadactyl individual.

Horses exhibit congruency in the metacarpal muscle attachments when compared to pentadactyl taxa [32,47], and retain muscles that normally attach to metacarpals I and V. Muscles such as abductor pollicis longus and extensor carpi ulnaris actually attach at their expected positions on reduced digits I and V, respectively, in the horse. However, this is probably due to consistency in the topological position of muscle precursors, rather than homology in digit identity [48].

Limb reduction has been extensively studied in squamates [49–51]. The progression of limb reduction in squamates follows a slightly different pattern from that in equids. In squamates, digit I is lost earliest in evolution, followed by digits V, II and III [49,52]. In equids, I is also the first digit lost, followed by V, II/IV and III persists as the dominant digit. During limb development, digits I and V are last to form, and therefore most likely to be anomalous or absent [25]. Correspondingly, we find that digits I and V are the least expressed proximally in the horse manus.

We find the horse distal forelimb shares features with that of an early horse embryo. Horse embryos at several stages (20, 50, 350 mm) show one, long, cylindrical dominant digit and two splint metacarpal digits, which commence as cartilaginous precursors that ultimately ossify [53]. Skeletal precursors are evident for digits II, III and IV, and are apparently absent for digits I and V; areas of apoptosis have been visualized surrounding digits II and IV [22]. In none of the described embryonic specimens does the horse limb commence as a flattened paddle that characterizes early limb development in humans and other species [54,55]; the horse limb at early stages already resembles the adult form. Pigs are artiodactyls that lack digit I. Pigs, unlike horses, exhibit cartilaginous precursors for all five digits during development, and the reduced digit has a smaller precursor that ultimately regresses [56]. The position of the digits in the horse remains unchanged from fetus to adult. Our fetal hoof specimens show four folds on the ventral surface, representing digits I, II, IV and V, and a smooth surface dorsally which represents digit III (figures 7*d–f* and 11*f–h*). In the adult, our proposed position of these digits remains ventral to digit III both proximally at the wrist and distally towards the distal phalanx, retaining their embryonic relations.

## 5. Conclusion

The morphology of the distal forelimb in the horse suggests an altered paradigm of monodactyly, in which remnants of the additional four digits are present both proximally and distally. The dominant digit remains as III. The splint metacarpals, previously interpreted as reduced digits II and IV, also contain remnants of digits I and V compressed as ridges onto the ventral surface. Our proposed locations of the proximal metacarpals are confirmed by their carpal articulations, which are consistent with those of the pentadactyl *Phenacodus*. The tridactyl equid *Mesohippus* shares the ventral ridges on the side digit bones, many of which appear to have a distinct fusion line, and exhibits a lesser degree of digit reduction than previously thought. The frog is a double structure unique to the modern horse, which is newly theorized to represent partially differentiated distal digits II and IV. The wings and hoof cartilages of the distal phalanx represent distal digits I and V. The frog and hoof wings each exhibit regions on the hoof with distinct keratinous lamellar fibres and germinating properties, further supporting our proposal that they represent additional digits. We reinterpret the famous *Hipparion* Laetoli footprints as exhibiting side expansions of the hoof wings (proposed digits I and V), rather than impressions from the complete side digits II and IV, and suggest the presence of a median claw in extinct equids, explaining the midline cleft on their distal phalanges. The nerves and arteries are more numerous than expected for a true mondactyl taxon, and better reflect the pentadactyl forefoot anatomy. Our study shows anatomical evidence for the presence of digits I and V proximally, in addition to the previously interpreted digits II, III and IV in the horse manus. In addition, we find remnants of digits I, II, IV and V distally surrounding dominant digit III. Our study suggests evidence of pentadactyly hidden within the single complete digit of *Equus*.

## Supplementary Material

Supplemental material

## Data accessibility

All data are provided within the text and figures of this article.
