# Supplementary material for: The evolution and anatomy of the horse manus with an emphasis on digit reduction
Source: R Soc Open Sci. 2018 Jan 24;5(1):171782. doi: 10.1098/rsos.171782 (PMC5792948; doi:10.1098/rsos.171782)
Supplement: Supplemental material [file rsos171782supp1.docx]

Materials: Specimen numbers

**Institutional abbreviations**

AMNH- American Museum of Natural History (New York, USA)

YPM- Yale Peabody Museum (New Haven, USA)

NS- Nikos Solounias personal collection

MCZ- Museum of Comparative Zoology (Cambridge, USA)

| **Taxon** | **Type of specimen** | **Specimen** |
| --- | --- | --- |
| *Equus* | skeletal | NS 273, AMNH 204176, AMNH 204165, AMNH 204159, AMNH 204143, AMNH 204144, AMNH 204115, AMNH 204089, AMNH 204110, AMNH 204111, AMNH 204108, AMNH 204114, AMNH 204117, AMNH 204112, AMNH 204109, AMNH 204093, AMNH 204183, AMNH 204186, AMNH 204193, AMNH 204028, AMNH 14132, AMNH 14131,AMNH 20402, AMNH 204190, AMNH 204210, AMNH 238322, AMNH 14131 |
| *Equus* | anatomic | NS 290, NS 291: fetal hooves used for histological sections counting nerves and vessels  NS 292: fetal hoof specimen photographed in Figures 7 and 11.  NS 296: late term hoof specimen photographed and sectioned in Figure 8.  NS 297: adult hoof specimen used for gross morphology of frog and keratinous hoof |
| *Dinohippus* | skeletal | AMNH 17224, AMNH FAM 87203, AMNH 116135, AMNH GY 2640, AMNH GY 1013712, AMNH GY 772767, AMNH GY 591759, AMNH GY 29912, AMNH GY 331283, AMNH GY 19396, AMNH GY 1394680, AMNH GY 1284434, AMNH GY1284437, AMNH GY1504927, AMNH GY 1484906, AMNH GY 1284438 |
| *Mesohippus* | skeletal | YPM 11312, YPM 1310, AMNH 1195, AMNH 705, AMNH 1189, AMNH 2454, AMNH 39001, AMNH 39480a, AMNH 39480b, AMNH 38858, AMNH 673, AMNH 1240, AMNH FAM 74025, AMNH 1477 |
| *Hyracotherium* | skeletal | AMNH 15428, AMNH 4832, YPM 11632, MCZ VPM-3440 |
| *Phenacodus* | skeletal | AMNH 4369, AMNH 2961 |
| *Hipparion* | skeletal | AMNH 109625, AMNH 10436, AMNH 10435, AMNH 109755, AMNH 109756, AMNH 98064, AMNH 109630, AMNH 10737, AMNH 23062, AMNH 23056, AMNH 23054, AMNH 20792, AMNH 22890 |
| *Hypohippus* | Skeletal | YPM 11623, AMNH 9407 |

Complete skeletal descriptions (carpal, metacarpal, phalanges)

***Hyracotherium***

The distal forelimb consists of four complete digits. Metacarpal III is the widest and longest bone. There is an elevated point on the lateral proximal articular surface of metacarpal III. The distal end of metacarpal III is slightly wider and fuller than the shaft. Metacarpals II, IV, and V are similar in shape, width, and length, with metacarpal IV being slightly larger. On the ventral surface of metacarpal II, there is a small triangular expansion proximally that tapers distally onto a thin, faint ridge that extends onto the ventral shaft. There are no ridges on the ventral surfaces of metacarpals III-V when articulated.

The four metacarpals are approximated towards the wrist, and they splay outward distally towards the phalanges. The four metacarpals have notably flattened dorsal surfaces that become slightly rounder and fuller towards the distal ends of the bones. There is an elongated central depression on the ventral surfaces of all four metacarpals. The keels of the distal condyles are visible on the ventral surfaces, but not the dorsal surfaces of the metacarpals.

The phalanges of all four digits are similar in length and width, with III being slightly largest.

The proximal phalanges are broad at the proximal articulations and narrow towards the distal articulations. The middle phalanges of digits II and III are shorter than those of IV and V. The distal end of the middle phalanx of III bulges dorsally.

The distal phalanx is tubular and elongated, with a notably arched dorsal surface. The dorsal surface is separated into two distinct areas: a rough, textured, pitted surface distally towards the terminal edge and a smooth surface proximally towards the middle phalanx. The extensor tubercle is blunted and not very prominent. There is an elongated, distinct median cleft on the terminal edge of the phalanges. The terminal edge is spade-shaped. The angles of the distal phalanges are notably blunted, and there are no wings.

***Mesohippus***

The forelimb consists of three digits (II, III, and IV), each with a metacarpal, proximal, middle, and distal phalanx. Metacarpal III is dominant; it is the largest and widest metacarpal. The proximal articular surface has an elevated point on the lateral aspect, corresponding to the articulation with the magnum and unciform. There is a large flattened plateau on the dorsal surface of the proximal shaft. The distal shaft flares slightly outward towards the proximal phalanx.

The articular view shows three distinct facets corresponding to metacarpals II, III, and IV, and large rounded protrusions over digits II and IV. Metacarpal II is more slender and slightly shorter than metacarpal III. There is an elevated ridge at the proximal shaft on the ventral side, which extends distally down the metacarpal. The ridge is distinct from the shaft of metacarpal II. Metacarpal IV is similar to metacarpal II in length and width. Metacarpal IV is notably thin proximally, widens at the mid-shaft, and again thins distally. In some specimens, metacarpal V presents as a short, proximal, tubular protrusion on the ventral surface of metacarpal IV. In specimens where this rudiment is absent, there is a distinct ridge with a fusion line on the ventral surface of metacarpal IV, which extends almost completely down the shaft. The ridges on the surfaces of metacarpals II and occasionally of IV of *Mesohippus* resemble those of the modern horse.

The phalanges of the dominant digit are larger, fuller, and wider than those of the side digits.

The dominant proximal phalanx has a broad proximal articulation. The mid-shaft is constricted and the distal articulation is narrower. The middle phalanx is characterized by wide proximal and narrower distal articulations. The dominant middle phalanx is notably short in relation to the proximal phalanx.

The dominant distal phalanx is a single, elongated, wedge-shaped bone. In dorsal view, the bone is somewhat narrow and V-shaped. The extensor tubercle is present and notably large in relation to the phalanx size. The dorsal surface of the distal phalanx is smooth proximal to the dorsal groove, but with fine ridges near the V-shaped edge. The terminal edge has a deep median split. The dorsal groove runs from the lateral edge of the bone to the median split.

The solar surface of the dominant distal phalanx is slightly concave. The flexor tubercle is well developed, and there are two depressions on either side of the tubercle. There is a constriction in solar view separating the distal phalanx proper from the angles. In lateral view, the distal phalanx is shallow, and there are no medial or lateral wings above the angles.

The side proximal and middle phalanges of digits II and IV are thin and complete. The side distal phalanges are long and curve inward. Laterally, each distal phalanx has a short blunted angle with no wing above it. The ventral surface has a large proximal protrusion. The dorsal groove is only visible on the inner surface. The ventral surface has numerous pits.

***Dinohippus***

We did not find carpal 1 associated with the specimens of *Dinohippus*, however the trapezoid appears to contain the facet for this bone, indicating that the trapezium was present.

The metacarpals appear to be isometrically smaller than those of *Equus*. There is one dominant central digit and two lateral splint bones. A rounded protrusion on the proximal aspect of the dorsal shaft of the dominant digit is present, and there is a distinct point at the proximal articular surface, separating the articulation with the unciform and the magnum. A wide flattened plateau exists proximally on the dominant digit ventrally between the splint bones. The dominant digit flares at the distal end before the condyle. The ventral surface of the dominant digit is flattened and the dorsal surface is rounded.

The splint bones are full proximally and taper distally. The splint bones are positioned laterally at the proximal edge, and are directed inwards towards the ventral midline of the shaft distally. On both the medial and lateral splint bones, there is a textured area at the proximal-ventral area. This textured surface extends distally into a thin ridge at the ventral surface, separating a small sliver of the splint bone from the remainder of the shaft. The distal aspect of the splint bones tapers into a thin knobby point. The ventral side of the distal articular surface of the digit III is narrow.

The phalanges are robust and one is dominant proximally, centrally, and distally. The proximal phalanx is long and has broad proximal and distal articulations. It is notably wide at the proximal and distal aspects and is thin centrally. The proximal aspect splays outward forming a “Y” with a depression in the center on the ventral surface. The middle phalanx is short and square shaped in dorsal and ventral views, but with wide proximal and distal articulations. The proximal surface of the middle phalanx is notably full and thick.

The distal phalanx is a wedge-shaped bone that appears crescentic in dorsal and solar views. The extensor process is low and broad. The dorsal surface possesses both a smooth area proximally and textured area with many grooves, foramina, and pits distally towards the crescentic edge. There is a small midline split on the terminal edge of the distal phalanx, which is most visible in dorsal view. The angles are present, but blunted. The wings are large, boxy, and, separated from the angles by a wide groove. The wings in dorsal view flare outward. The wings are separated into parietal and inner surfaces by a shallow groove. The dorsal groove terminates before the midline of the distal phalanx.

***Equus***

The proximal row of carpals consists of 4 bones. From medial to lateral, they include the scaphoid, lunate, triquetrum, and pisiform. The distal row of carpals contains three or four bones; from medial to lateral this includes the trapezoid, magnum, and unciform (carpals 2-4 respectively). The trapezium (carpal 1) is variably present.

The metacarpal region consists of a dominant central bone and two lateral splint bones, which are often partially fused to the main digit. The dominant metacarpal is flattened on the ventral side and rounded on the dorsal side. There is a point at the proximal articular surface, corresponding to the junction of the unciform and magnum articulations. The distal condyle has a median keel that extends onto both the dorsal and ventral surfaces.

The medial splint bone has an elevated proximal area that continues distally into a thin ridge that extends the majority of the length of the bone. The proximal area and ridge are textured and rough, and appear distinct from the smooth surface of the splint bone in lateral view. Distally the medial splint bone flares outward and terminates in a large, flattened knob. There is an expansion of the distal medial splint bone onto the surface of the dominant digit. The lateral splint bone is rounded proximally. There is a large, elevated plateau with a textured surface at the proximal splint bone, which extends distally down the shaft. The lateral splint bone is smaller and thinner than the medial splint bone, and it terminates in a small, rounded knob. Neither splint bone ends in a synovial joint.

Ventral to the metacarpophalangeal joint (fetlock), there are two large sesamoids, termed the proximal sesamoids. There are three phalanges on the functional manus: proximal (long pastern), middle (short pastern), and distal (coffin). Between the middle and distal phalanges there is a rhomboid-shaped bone called the distal sesamoid. In colloquial equestrian terminology the distal sesamoid is often referred to as the navicular; not to be confused with the true navicular of the foot.

The proximal phalanx is long and has a broad proximal articulation. The mid-shaft is slightly constricted. The scar for the attachments of the sesamoid ligaments is V-shaped with a central flattened area and a rounded distal surface. The middle phalanx is characterized by equally wide proximal and distal articulations. The scar for the tendon of the flexor digitorum superficialis is located at the proximal surface of the phalanx.

The distal phalanx (coffin bone) is single and wedge-shaped with a crescentic terminal edge. The crescent is smooth, without a median split, and forms a broad, uninterrupted, symmetrical “U,” creating the characteristic horseshoe shape. The bone near the crescent edge is perforated with multiple foramina and covered by fine ridges from the keratinous hoof. The extensor process is large, V-shaped, and is positioned at the median plane in dorsal view. In solar view, the crescentic surface terminates in two symmetrical pointed knobs, termed the angles, with one medial and the other lateral. Near the coffin joint on the solar surface, the attachment for the flexor digitorum profundus forms the flexor tubercle.

Above the angles there are two symmetrical dorsal protrusions named the wings, which are positioned medially and laterally. The wings are triangular/wedge-shaped and are pointed dorsally, paralleling the shape and orientation of the extensor tubercle. In dorsal view, the wings are oriented vertically. The size of the wings varies between individuals. The wings are separated into a parietal and inner surface by a deep groove, which houses a portion of the medial and lateral hoof cartilages. The parietal surface of each wing is flat and the inner surface is notably rough and pitted. The hoof cartilages protrude behind the distal phalanx, and on the internal aspect, there are numerous ligaments. There is a large foramen between each angle and wing (one medial and one lateral).
